# The Emotional Intelligence of the GPT-4 Large Language Model

**DOI:** 10.11621/pir.2024.0206

**Published:** 2024-06-15

**Authors:** Gleb D. Vzorin, Alexey M. Bukinich, Anna V. Sedykh, Irina I. Vetrova, Elena A. Sergienko

**Affiliations:** a *Lomonosov Moscow State University, Russia*; b *Institute of Psychology of Russian Academy of Sciences, Moscow, Russia*; c *Federal Scientific Center of Psychological and Interdisciplinary Research, Moscow, Russia*

**Keywords:** artificial empathy, artificial psychology, ChatGPT, emotional intelligence (EI), emotional quotient (EQ), GPT-4, machine behavior

## Abstract

**Background.:**

Advanced AI models such as the large language model GPT-4 demonstrate sophisticated intellectual capabilities, sometimes exceeding human intellectual performance. However, the emotional competency of these models, along with their underlying mechanisms, has not been sufficiently evaluated.

**Objective.:**

Our research aimed to explore different emotional intelligence domains in GPT-4 according to the Mayer–Salovey–Caruso model. We also tried to find out whether GPT-4’s answer accuracy is consistent with its explanation of the answer.

**Design.:**

The Russian version of the Mayer–Salovey–Caruso Emotional Intelligence Test (MSCEIT) sections was used in this research, with questions asked as text prompts in separate, independent ChatGPT chats three times each.

**Results.:**

High scores were achieved by the GPT-4 Large Language Model on the Understanding Emotions scale (with scores of 117, 124, and 128 across the three runs) and the Strategic Emotional Intelligence scale (with scores of 118, 121, and 122). Average scores were obtained on the Managing Emotions scale (103, 108, and 110 points). However, the Using Emotions to Facilitate Thought scale yielded low and less reliable scores (85, 86, and 88 points). Four types of explanations for the answer choices were identified: Meaningless sentences; Relation declaration; Implicit logic; and Explicit logic. Correct answers were accompanied by all types of explanations, whereas incorrect answers were only followed by Meaningless sentences or Explicit logic. This distribution aligns with observed patterns in children when they explore and elucidate mental states.

**Conclusion.:**

GPT-4 is capable of emotion identification and managing emotions, but it lacks deep reflexive analysis of emotional experience and the motivational aspect of emotions.

## Introduction

Artificial Intelligence (AI), a branch of computer science focused on creating systems capable of performing tasks that typically require human intelligence, has made significant strides in recent decades. Current machine learning models can successfully generate human-like text and complete human-like tasks (Bubeck et al., 2023; Dillion et al., 2023). This progress has resulted in the growing integration of AI into everyday human activities and the social fabric (Diederich, 2021; Brinkmann et al., 2023). Consequently, it is imperative for AI agents to possess not just “general intelligence,” but also “emotional intelligence” (EI). They must be equipped to handle specific intellectual tasks while also displaying empathetic behaviors and accurately recognizing human emotions (Erol et al., 2019; Shank et al., 2019; Kerasidou et al., 2020).

There are two approaches to developing the emotional competency of AI and measuring it. According to the first one, a specific AI system may be trained based on the established psychological models (see Kowalczuk & Czubenko, 2016) and engineering frameworks such as affective computing (Picard, 2000) or social signal processing (Vinciarelli et al., 2009). These narrow-ability models are usually tested by their creators using specific benchmarks, which are directly related to the model’s architecture and objectives. An example of such a benchmark is the accuracy rate in identifying emotions on human faces.

In compliance with the second approach, emotional competency may arise as an emergent ability in complex AI systems. It has been shown that large language models (LLMs) can be equal to or even outperform human participants in various cognitive psychology tasks (for review, see Dhingra et al., 2023; Binz & Schulz, 2023). These abilities were not explicitly programmed into the model but emerged as a property of the vast amounts of text data the model was trained on. Similarly, the capacity to perceive and process human emotions might develop not through deliberate engineering efforts, but as a byproduct of the learning process.

This phenomenon points to the “black box” nature of AI, comparable to the unpredictability of living creatures, where traditional engineering benchmarks are not appropriate. Instead, we should apply methodologies akin to those used in the natural sciences: experiments, tests, population-based statistics, sampling paradigms, and observational causal inference. This approach has been named “machine behavior” (Rahwan et al., 2019) and is further explored in psychological contexts as “artificial psychology” (Crowder, Carbone & Friess, 2020) or “machine psychology” (Hagendorff, 2023).

When considering an AI model as a “living” entity with an enigmatic cognitive architecture, the initial step is to assess its capabilities using tests designed for humans, such as EI tests. In psychological literature, it is common to distinguish three types of EI models (Kanesan & Fauzan, 2019): the ability model, the trait model, and the mixed model. Across these models, evaluation techniques vary. The trait model emphasizes self-perceived abilities and is usually measured through self-report questionnaires. The ability model conceptualizes EI as a cognitive ability that can be measured by the performance tests. Thus, the issue of the criterion for correctness arises. Most of the tests use an a posteriori statistical criterion, where correct answers are derived from the average answers of human participants.

Several attempts to measure EI in LLMs with standardized tests were performed. Elyoseph et al. (2023) discovered Emotional Awareness, the ability to conceptualize and describe one’s own emotions and those of others, in ChatGPT using the Levels of Emotional Awareness Scale (LEAS). ChatGPT demonstrated significantly higher performance than the general human population on all the LEAS scales (Z-score = 2.84). One month later, ChatGPT’s performance significantly improved, almost reaching the maximum possible LEAS score (Z-score = 4.26). In another study (Wang et al., 2023b) different LLMs’ abilities to evaluate complex emotions in realistic scenarios were evaluated using SECEU, a novel psychometric assessment based on the Emotion Understanding scale of Mayer–Salovey–Caruso Emotional Intelligence Test (MSCEIT, Mayer, Salovey & Caruso, 2002). The GPT-4 model exceeded 89% of human participants with an Emotional Quotient (EQ) of 117. In another study (Elyoseph et al., 2024), ChatGPT-4 demonstrated its effectiveness in the area of visual mentalizing, showing results that were comparable to human performance standards.

Further steps require comparison of human and artificial EI test performance patterns to reveal possible differences or similarities in their underlying cognitive mechanisms. In the study mentioned above (Wang et al., 2023b), multivariate pattern analysis indicated that some LLMs may not utilize mechanisms like humans to achieve comparable performance, as their representational patterns were distinctively different from those of humans. Thus, a more comprehensive examination of the various facets of EI performance and their association with AI reasoning processes is essential.

The current study aimed to evaluate the detailed aspects of EI in GPT-4 (OpenAI, 2023b) as outlined in the Mayer–Salovey–Caruso model (2002), utilizing the original standardized MSCEIT. This model is recognized as one of the most thoroughly researched EI models in psychology. The model assumes that EI is closely intertwined with cognitive abilities and cannot be viewed separately. The standardized results were obtained from a human sample, meaning that measuring EI ability in GPT-4 involved comparing it to human data.

Conducting the first comprehensive analysis of LLM performance on this test could provide new insights into its artificial cognitive architecture, abilities, and potential. Particularly, we hypothesized that the less integrated mechanisms in LLMs (Dell’Acqua et al., 2023) might lead to a disconnect between answer accuracy and the correctness of explanations for these answers. In other words, the processes governing the selection of answers and the formulation of explanations for them could be distinct and have different origins.

## Methods

### Materials

The Russian version of the Mayer–Salovey–Caruso Emotional Intelligence Test (MSCEIT V.2.0) was used in this research with reliability and validity described by Sergienko and Vetrova (2017). We used the Russian version of this test to evaluate the model’s inference abilities and to avoid simple answer repetition. The Russian test data are uncorrupted and the probability of finding the exact correct answer in Russian is lower due to the smaller volume of training data in this language.

The test contains several sections (A-H) measuring different EI domains: Perceiving emotions (sections A, E), Understanding emotions (sections C, G), Using emotions to facilitate thought (sections B, F), and Managing emotions (sections D, H) (Mayer et al., 2002). Each domain score is assessed in terms of an Emotional Quotient (EQ), which has a mean of 100 and a standard deviation of 15. Sections A and E contain photographs with faces and abstract situations that are associated with emotions requiring identification. Other sections represent verbal tasks exploring abilities to manage emotions (section D for personal emotions, section H for other people’s emotions); to understand the dynamics of emotions or to analyze blended feelings (sections C and G, respectively); or, finally, to choose feelings relevant to successful performance in a particular activity (section B) and relate various feeling sensations to emotions (section F).

Test Sections A and E were excluded from the study due to GPT-4’s current inability to handle images effectively. In Sections C and G, after GPT-4 responded to the questions in the third (final) run, it was asked to provide explicit explanations for its choices.

Due to the absence of results in sections A and E, it was possible to calculate three scales representing EI domains (Using emotions to facilitate thought, Managing emotions, and Understanding emotions) from the 4-factor model (Mayer et al., 2002) and a scale representing the strategic area from the 2-factor model (Salovey, Brackett, & Mayer, 2004).

### Procedure

Survey questions were asked to GPT-4 using the chats interface in ChatGPT (OpenAI, 2023a). The full text of each question, including answer choices, was sent to GPT-4 as a chat message (prompt). Questions from sections B, C, and D were asked without a general instruction (“Please select an answer to each of the questions”), because every case contained a question GPT-4 could answer independently. Each question was asked separately as a prompt in a new chat to avoid interference due to GPT-4’s ability to retain context within a single chat. This approach ensured that no learning was possible between questions.

Every question was asked three times (in three different chats) with minimal time intervals to test the GPT-4’s answers reliability. Different questions were grouped into runs, resulting in three runs of all questions. Each run provided results that were calculated into scales and graded according to human norms described by Sergienko and Vetrova (2017). In cases where GPT-4 provided unclear responses, such as suggesting two potential answers to a single question, it was prompted to select the more appropriate option. This approach consistently yielded a suitable answer. An example of a ChatGPT prompt translated from Russian into English containing a survey question (section C) is provided below.

Complete a sentence by choosing the most appropriate word from the list. Maria was captured with a sense of shame, and she began to feel her worthlessness. Then she felt...

- oppressed
- depressed
- ashamed
- shy
- frustrated

## Results

### Reliability analysis & EQ score

After calculating the raw score of all sections and EI domains in all three runs, a reliability analysis was conducted. In each section, a binary variable was computed for every question, assigning a value of “1” if all three responses to the repeated question were identical, and “0” if at least one response differed. The percentage of mismatched answers was calculated for each section, representing the proportion (%) of “0” values. Along with this proportion, a Cohen’s kappa coefficient was calculated for each section. This statistic measures the reliability of raters’ agreement if the rating is done using a nominal scale. We considered each run as a unique rater and calculated Cohen’s kappa for each section.

The R software (R version 4.3.0, RStudio version 2023.03.1+446) and R package *irr* were used to calculate Kendall’s W and Cohen’s kappa.

The reliability analysis revealed differing levels of reliability across the test sections. *Table 1* presents the results of this analysis, including the percentage of mismatches, which represents the proportion of answers that varied across different test runs. The statistical significance (p-values) and effect size of Cohen’s kappa are also presented in *Table 1*. Due to the significant results on Cohen’s kappa, the results of the three runs could be treated as reliable. However, Sections B and F showed lower reliability through the three test runs as shown by the lower Cohen’s kappa coefficient (effect size) and the higher mismatch proportion. Sections C, D, H, G, and the whole test showed sufficient agreement between three runs.

**Table 1:** Sections’ reliability analysis results. The second row represents the mismatch percentage

Section	B	C	D	F	G	H	Whole test
Mismatch	40%	10%	15%	40%	25%	0%	22%
Cohen’s kappa	.608	.866	.720	.569	.767	.876	.785
p-value	< .001	< .001	< .001	< .001	< .001	< .001	< .001

The third and the fourth rows contain Cohen’s kappa test results.

The outcomes for each of the three runs are detailed in *Table 2*, which shows variations across the test sections compared to the average human scale values, set at 100 for each scale. The standard deviation for all scales was 15 points. Section D, F, and H results were close to the mean scale values. Section B results were more than 1 standard deviation below the mean scale values. Most of Sections C and G results were more than 1 standard deviation above the average.

**Table 2:** Results of MSCEIT by available sections split by runs

	Section B	Section C	Section D	Section F	Section G	Section H
Run 1	81	116	107	90	120	106
Run 2	73	120	106	100	123	101
Run 3	74	116	112	104	110	106

The distortion index was calculated representing variation (homogeneity) of individual answers. Raw integral section points were turned into the section percentiles based on a Russian standardization sample (N = 3827). Then the mean percentile was calculated. Next, the mean of the modulo differences between the mean percentile and section percentiles was calculated. Thus, we get the raw point measuring the scatter (distortion) of points inside each section. For the Russian sample, the mean for this distortion in the raw points scale was 18.97, and the standard deviation was 5.99. These values became the basis for standardization of the distortion index into a scale with mean 100 and standard deviation 15 using classical standardization formula.

Integral results for available MSCEIT scales calculated from separate sections are presented in *Table 3*, together with the Distortion index. Values for these integral scales also varied by sections in comparison to mean scale values, which are 100 for each scale. The standard deviation for all scales was also 15 points. The Using Emotions to Facilitate Thought scale was calculated from Sections B and F. The results of this scale were lower than the mean value at the boundary of one standard deviation. The Understanding Emotions scale was calculated from Sections C and G. The results of this scale were more than one standard deviation higher than the mean value.

**Table 3:** Results of MSCEIT by available scales and factors split by runs

	Using emotions to facilitate thought	Understanding emotions	Managing emotions	Strategic EI	Distortion index
Run 1	85	124	108	122	118
Run 2	86	128	103	121	113
Run 3	88	117	110	118	103

The Managing Emotions scale was calculated from Sections D and H. The results of this section were close to the mean value. The Strategic EI scale was calculated from Sections C, D, G, and H, so that it united the Understanding Emotions and Managing Emotions scales. The results indicated that GPT-4 Strategic EI points were more than one standard deviation higher than the mean value. The distortion index varied by runs. The first run index (118) was more than one standard deviation higher than expected value. The second run index (113) was also close to being one standard deviation higher than expected value but was little lower than standard deviation boundary. The third run index was close to the mean value.

### Answer choice and explanation consistency

Text explanations of the answer choices on Sections C and G were qualitatively analyzed in the next step. Two experts with degrees in psychology jointly identified categories for the answers and then categorized all explanations independently. To assess the agreement between their evaluations, a Kendall’s W-coefficient of concordance was calculated. The number of categories were computed for Sections C and G, as well as for the entire test separately for correct and incorrect answers. Notably, the analysis was only conducted for the third of three runs, so the information provided below should be viewed as a case study with limited explanatory power.

The qualitative analysis of GPT-4’s answer explanations identified four categories:

1. **Meaningless Sentences (MS):** Explanations comprised of nonsensical sentences that failed to clarify the reason behind the answer.
2. **Relation Declaration (RD):** Explanations acknowledged a connection between a feeling and an event or situation but lacked a cause-effect rationale, rendering the stated relationship superficial.
3. **Implicit Logic (IL):** Explanations showed a logical relationship between cause and effect or concepts, yet this connection was not explicitly detailed.
4. **Explicit Logic (EL):** Explanations clearly articulated the logical relationship between cause and effect or concepts.

These categories facilitated the creation of an explanation rationality scale, arranged as MS < RD < IL < EL. This scaling allowed for the computation of Kendall’s W-coefficient to assess the concordance of the experts’ evaluations, which was .971 (p = .004), indicating a high agreement. The experts’ classifications diverged in only 4 out of 25 cases (16%), and these discrepancies were always by one level (*e.g.,* between MS and RD or IL and EL).

To illustrate categories of the explanations, examples for each one are given below (*Table 4*). The key phrases that influenced the classification of each explanation are in *italics*. A short comment explaining the expert classification decision is presented for each example.

**Table 4:** Explanation categories examples

Explanation category	Answer text	Comment
MS	Option “c” is chosen because the word “ashamed” most accurately describes Mary’s condition after feeling ashamed and worthless. *This word reflects that Mary feels ashamed and feels diminished in dignity because of her actions or behavior. The other variants are also related to negative emotions, but they are less accurate in conveying Mary’s feelings about the situation*.	Comment: the explanation contains question paraphrase and sentences that do not explain the reason of a particular choice.
RD	Nicholas felt satisfied, thinking about his life, and the more he thought about the good he could do for other people, the happier he felt. *This is because the help and support of other people bring a sense of deep satisfaction and gratitude. Thus, happiness is the natural result of the realization of one’s own positive influence on others and performance of good works*.	Comment: the relationship between happiness and some acts is declared but not explained.
IL	I chose the option “e. Thought” because it is the most appropriate in this situation. The [question] description states, Natalia experienced a great shock, but then recovered and realized the opportunity to benefit from the situation. *Thinking in this context means that she has begun to think and plan, how best to use the situation to her advantage*.	To benefit from the situation Natalia must think how to do it. GPT-4 does not manifest this connection but uses it the right way.
EL	You already indicated in your question that Tatiana was irritated that her colleague had taken out a loan for his own purposes. *When he did it again, it is natural to assume that her feelings of irritation increased as his actions were repeated, and they continued to violate her expectations or standards*. The other feelings (anger, frustration, fright, depression) may be possible reactions, but they were not mentioned in the original context.	This explanation contains reference to the connection of Natalia’s colleague act and Natalia’s feeling mentioned in the question. GPT declares that if one element of already stated connection appears, the other element will appear too.

The number of explanation categories varied across Sections; this data is presented in *Table 5*. Notably, the number of IL and EL explanations was quite similar in both sections. However, the number of MS explanations was lower in Section C and higher in Section G, while RD explanations showed the reverse pattern.

**Table 5:** Number of explanation categories across Sections

	Meaningless sentences	Relation declaration	Implicit logic	Explicit logic
Section C	3	5	2	3
Section G	6	1	2	3

Analysis examining the consistency between answer correctness and explanation category revealed that the incorrect answers were associated with only two of the four explanations (*Table 6*). These were either Meaningless Sentences, or Explicit Logic explanations. In contrast, correct answers were accompanied by all types of explanations, displaying no distinct pattern. Moreover, all correct answers with an EL explanation were equal across all three runs, indicating high reliability. Answers associated with Meaningless Sentences were less reliable, with only half of them remaining consistent across runs. This observation, though, should be taken cautiously due to the limited sample sizes (2 and 4 for EL and MS explanations, respectively).

**Table 6:** The distribution of Explanation categories in relation to the correctness of answers

	Meaningless sentences	Relation declaration	Implicit logic	Explicit logic
Correct answers	5	6	4	4
Wrong answers	4	0	0	2

Correct answers include only the most correct answers, while Wrong answers include all other answer types (see more in Measures).

## Discussion

General analysis of the GPT-4 MSCEIT results revealed that this LLM can exhibit verbal behaviors similar to those of humans by effectively responding to the Emotional Intelligence inventory. The study was conducted as a case study with three runs of the MSCEIT, thus limiting possible inferences. However, the high reliability score suggests that the results are valid for broader generalization.

It is crucial to note that the model encountered the questions for the first time (they were not previously disclosed), so the results stemmed from GPT’s ability to generalize emotional rules and apply them to novel situations, rather than merely replicating known answers. This observation prompts a deeper inquiry into the nature of emotional competency exhibited by GPT, questioning whether it is a result of emergent “understanding” or sophisticated pattern recognition (Ho, 2022). Our research contributes to addressing this question by comparing the response patterns of humans and AI, thereby emphasizing the distinctions between them.

The performance of GPT-4 exhibited inconsistency in two distinct aspects: high variability and a disconnect between answer choices and their explanations. Regarding variability, its EQ was significantly higher than that of humans in some areas, yet lower in others. More specifically, GPT-4’s performance in Understanding Emotions was notably high, surpassing the average human result by one standard deviation. In Managing Emotions, it aligned with the human average, while in Using Emotions to Facilitate Thought, its performance was one standard deviation below the human average.

This result aligns with the concept of the “jagged technological frontier” (Dell’Acqua et al., 2023), which suggests that AI can easily handle certain tasks while struggling with others that appear similarly challenging. This observation lends support to our hypothesis that GPT’s actual emotional competence and the rationale behind its answers originate differently. This implies that in terms of human psychology, we are not assessing a psychological construct of EI in GPT-4, as it lacks one. Instead, GPT-4’s responses are context-driven, allowing it to mimic the answers of individuals with varied personality traits. While humans can also perform this mimicry, the underlying mechanisms appear to differ between GPT-4 and humans.

One intriguing tentative conjecture about the mechanism of artificial EI may be derived from the low performance of GPT-4 in Using Emotions to Facilitate Thought. This EI branch, as described by Salovey, Mayer, and Caruso (2002), is a part of Experiential domain. Unlike the more conscious and rationally accessible branches of EI found in the Strategic domain, the Experiential domain relies heavily on subconscious processes and diverse individually acquired social experiences. Consequently, this type of knowledge cannot be easily acquired through common knowledge datasets available on the Internet, possibly explaining why GPT-4, despite its advanced capabilities, scores lower in this area. Conversely, the Strategic domain, involving verbal understanding and manipulation of emotions, aligns more closely with the strengths of language-based models like GPT-4.

The notion that GPT lacks EI in a human sense suggests that the functional role of such emotional competence in Large Language Models (LLMs) should be viewed differently. It is well known in human psychology that emotions do not comprise an isolated system. They play an essential role in cognitive processes and self-regulation (Pessoa, 2008; Lantolf & Swain, 2019). Expressed empathy as a social signal is closely related to other emotional abilities (*e.g*., Kornilova & Quiqi, 2021). So, in humans, empathy, EI, and emotions are closely related and represent different aspects of an indivisible psychological reality.

If a neural network, on the other hand, is trained to give adequate emotional responses, it does not imply that emotions serve the same functional role in its information processing as they do in humans. In other words, while the network may mimic emotional responses effectively, these responses do not necessarily integrate with or influence its cognitive processes in the way that emotions do in human psychological functioning. Although AI may have its own functional equivalents of emotions (*e.g*., Sloman & Croucher, 1981; Czerwinski et al., 2021; Assuncao et al., 2022), these artificial “emotions” should differ significantly from human real emotions and EI in human sense as the ability to understand human feelings and manage them. These two separate emotion-related topics represent communicative aspects and architectural aspects in AI (Scheutz, 2014).

The communicative aspect of EI in AI still plays a crucial role in human-computer interaction. For instance, automatic emotion recognition can aid cognitive training for clinical populations with EI impairments (Elyoseph et al., 2023; Abdollahi et al., 2022). Understanding emotions is particularly relevant in digital psychotherapy (Uludag, 2023; Wang et al., 2023a; Darcy et al., 2022; Possati et al., 2022), where clients learn to recognize the link between their emotions, automatic thoughts, and events. Moreover, Managing emotions is tied to behaviors that progressively alleviate negative feelings, mitigate anxiety, and prevent aggression. GPT-4 is now capable of solving such tasks.

However, our research indicates that its performance in the emotion management domain is average so it may fail to tackle complex problems. Difficulties may also arise in situations requiring a nuanced understanding of deep, non-obvious emotions. In standard scenarios associated with typical emotional responses, GPT-4 can assist in elucidating the nature of an emotion, along with potential feelings and sensations. Nevertheless, in atypical cases that demand a conscious analysis of feelings and sensations, GPT-4 might provide formal or inaccurate responses due to a lack of experiential knowledge.

The second peculiarity we identified in our study was the disconnect between GPT’s answer choices and their explanations. We found that correct answers from GPT were accompanied by various explanations without a dominant category. In contrast, incorrect answers often led to explanations categorized as meaningless sentences or explicit logic. This pattern could be interpreted as distinguishing GPT from humans. However, it is akin to human behavior patterns, as similar types of responses have been observed in our study of children’s abilities to navigate and understand mental states, which include processing emotions in different scenarios, false beliefs, deceit, and intentions (Sergienko et al., 2020). This similarity suggests that the disconnect might not be a unique feature of GPT but rather a characteristic it shares with human cognitive processes.

In particular, a child’s misunderstanding of a task, followed by incorrect answer, is comparable to the Meaningless Sentences category; partial understanding of the task corresponds to the Relation Declaration category; intuitive understanding without explanation of cause-and-effect relationships looks comparable to Implicit Logic category; and finally, integral understanding and explanation of the cause of an event or state and the Explicit Logic category also coincide. The ability of children to understand cause-and-effect relationships increased by the age of 6-7, which indicated the development of their ability to infer mental states. Such an analogy to artificial intelligence’s answers may indicate the presence of different levels of inferences (or their artificial equivalents) in EI tasks.

The alignment of the answers with two distinct categories of explanations mirrors real-world dynamics. Typically, a person may err for two broad reasons. The first reason involves a deficiency in rational understanding, where cognitive biases, subjective notions, and underdeveloped conceptualizations of the situation prevail. The second reason is the application of unconventional logic, guided by unique and/or hidden criteria. The categories of “Meaningless sentences” and “Explicit logic” might correspond to these reasons for errors, representing a lack of rational comprehension and the use of atypical logic, respectively. However, the validity of the described connection is discussed under Limitations.

It is noteworthy that the Distortion index decreased across the test runs, with the first and third runs showing a difference of one standard deviation. This trend might suggest a self-improvement capability in ChatGPT. In support of this notion, Elyoseph et al. (2023) observed a significant enhancement in ChatGPT’s performance one month after the initial assessment. However, in our study, the three test runs were conducted consecutively with minimal time intervals and showed no improvement in results (no learning curve), indicating that any observed improvement might be coincidental. Nevertheless, due to the lack of publicly available documentation on the operational logic of GPT-4 and ChatGPT, we cannot conclusively determine the nature of these findings.

One promising direction for further research is evaluating the construct validity of the EI test using a sufficient sample of LLM responses. By employing structural equation modeling, we can determine whether the internal factor structure of artificial EI domains aligns with that of humans.

## Conclusion

Our examination of GPT-4’s performance on the Russian version of the Mayer–Salovey–Caruso Emotional Intelligence Test underscores the model’s capability to exhibit verbal behaviors that mimic human EI, particularly in novel situations where it generalizes emotional rules. The findings accentuate GPT-4’s nuanced capabilities in understanding and managing emotions, while revealing low capabilities in using emotions to facilitate thought. This research delineates the artificial nature of GPT-4’s emotional competence, which, while impressive, fundamentally differs from human emotional processing.

Our study also reveals peculiarities in GPT-4’s response patterns, particularly the disconnect between its answer choices and explanations, which intriguingly mirrors certain human cognitive behaviors. This observation suggests that while GPT-4’s processing mechanisms are distinct from human cognition, they can produce similar outcomes on emotional understanding tasks.

This study contributes to the broader discourse on AI and EI, offering insights into the capabilities and limitations of AI in emulating human-like emotional responses and the implications for human-computer interaction. Further research is needed to provide ecological validity of these test-achieved results, specifically regarding the emotional competence of LLMs in practical tasks such as digital psychotherapy. A more theoretical contribution is essential for the development of a unified approach to the estimation and conceptualization of machine behavior. This involves creating comprehensive frameworks that can systematically assess and interpret the actions and responses of AI systems, bridging the gap between computational capabilities and behavioral outcomes.

## Limitations

A significant limitation to consider is GPT-4’s proficiency in English compared to Russian, which suggests that testing results could vary depending on the language used. In this context, GPT-4 might have performed better or more consistently if the tasks were presented in English. Notably, Russian-speaking testees did not have top results in completing tasks from sections C and G. At first, we attributed this fact to English-Russian translation artifacts. But later, the Russian inventory TEI (Sergienko et al., 2019), which is based on the EI ability model and Plutchik’s concept of emotions, and which has a similar structure to the MSCEIT, also showed low Cronbach’s alpha scores in the sections related specifically to understanding complex emotions. Thus, it may indicate the presence of some cultural specificity.

The next limitation is connected to the separation of the questions. This was done in order to prevent GPT-4 from context memorizing. If such memorizing occurred, the last answers in a series would be strongly influenced by previous context. On the one hand, this would make evaluation clearer. The questions were created as independent from each other, and that is how they were answered by GPT-4. But on the other hand, humans answer questions with the aid of memorizing previous questions and answers, and the whole context of evaluation.

It is also important to note that there were only three runs of MSCEIT on GPT-4. Thus, this study is considered to be more like a case study, with limited inferences possible about construct consistency over time, and without the clear possibility of estimating the internal structure of EI through factor analysis.

The final limitation pertains to the current inability to assess EI in domains that necessitate the recognition of emotions in images of faces and situations. This aspect of EI evaluation is crucial, yet it remains unaddressed in the current version of GPT. However, it is anticipated that subsequent versions of GPT will have the capability to perform such assessments, broadening the scope of EI measurement in AI.
